# Neutralizing activity against Omicron BA.5 after tixagevimab/cilgavimab administration comparable to those after Omicron BA.1/BA.2 breakthrough infections

**DOI:** 10.3389/fimmu.2023.1139980

**Published:** 2023-03-02

**Authors:** Jinyoung Yang, Gunho Won, Jin Yang Baek, Young Ho Lee, Haein Kim, Kyungmin Huh, Sun Young Cho, Cheol-In Kang, Doo Ryeon Chung, Kyong Ran Peck, Kyo Won Lee, Jae Berm Park, Sang Eun Yoon, Seok Jin Kim, Won Seog Kim, Min Su Yim, Kwangwook Kim, Seokhwan Hyeon, Byung Chul Kim, Yoo-kyung Lee, Jae-Hoon Ko

**Affiliations:** ^1^ Division of Infectious Diseases, Department of Medicine, Samsung Medical Center, Sungkyunkwan University School of Medicine, Seoul, Republic of Korea; ^2^ Division of Vaccine Development Coordination, Center for Vaccine Research, National Institute of Infectious Diseases, National Institute of Health, Korea Disease Control and Prevention Agency, Cheongju, Republic of Korea; ^3^ Asia Pacific Foundation for Infectious Diseases (APFID), Seoul, Republic of Korea; ^4^ Division of Transplantation Surgery, Department of Surgery, Samsung Medical Center, Sungkyunkwan University School of Medicine, Seoul, Republic of Korea; ^5^ Division of Hematology-Oncology, Department of Medicine, Samsung Medical Center, Sungkyunkwan University School of Medicine, Seoul, Republic of Korea

**Keywords:** COVID-19, SARS-CoV-2, Evusheld, neutralizing antibody, BA.5 variant

## Abstract

**Introduction:**

The effect of tixagevimab/cilgavimab (Evusheld™; AstraZeneca, UK) should be evaluated in the context of concurrent outbreak situations.

**Methods:**

For serologic investigation of tixagevimab/cilgavimab during the BA.5 outbreak period, sera of immunocompromised (IC) hosts sampled before and one month after tixagevimab/cilgavimab administration and those of healthcare workers (HCWs) sampled one month after a 3^rd^ shot of COVID-19 vaccines, five months after BA.1/BA.2 breakthrough infection (BI), and one month after BA.5 BI were investigated. Semi-quantitative anti-spike protein antibody (Sab) test and plaque reduction neutralizing test (PRNT) against BA.5 were performed.

**Results:**

A total of 19 IC hosts (five received tixagevimab/cilgavimab 300 mg and 14 received 600 mg) and 41 HCWs (21 experienced BA.1/BA.2 BI and 20 experienced BA.5 BI) were evaluated. Baseline characteristics did not differ significantly between IC hosts and HCWs except for age and hypertension. Sab significantly increased after tixagevimab/cilgavimab administration (median 130.2 BAU/mL before tixagevimab/cilgavimab, 5,665.8 BAU/mL after 300 mg, and 10,217 BAU/mL after 600 mg; both *P* < 0.001). Sab of one month after the 3^rd^ shot (12,144.2 BAU/mL) or five months after BA.1/BA.2 BI (10,455.8 BAU/mL) were comparable with that of tixagevimab/cilgavimab 600 mg, while Sab of one month after BA.5 BI were significantly higher (22,216.0 BAU/mL; *P* < 0.001). BA.5 PRNT ND_50_ significantly increased after tixagevimab/cilgavimab administration (median ND_50_ 29.6 before tixagevimab/cilgavimab, 170.8 after 300 mg, and 298.5 after 600 mg; both *P* < 0.001). The ND_50_ after tixagevimab/cilgavimab 600 mg was comparable to those of five months after BA.1 BI (ND_50_ 200.9) while ND_50_ of one month after the 3^rd^ shot was significantly lower (ND_50_ 107.6; *P* = 0.019). The ND_50_ of one month after BA.5 BI (ND_50_ 1,272.5) was highest among tested groups, but statistical difference was not noticed with tixagevimab/cilgavimab 600 mg.

**Conclusion:**

Tixagevimab/cilgavimab provided a comparable neutralizing activity against the BA.5 with a healthy adult population who were vaccinated with a 3^rd^ shot and experienced BA.1/BA.2 BI.

## Introduction

Passive immunization using convalescent plasma or monoclonal antibodies has been widely studied since the coronavirus disease 2019 (COVID-19) pandemic began, but the narrow administration time window (effective only at the early course of illness), short half-life, and rapidly emerging severe acute respiratory syndrome coronavirus 2 (SARS-CoV-2) variants limited its clinical use (1–3). Tixagevimab/cilgavimab (Evusheld™; AstraZeneca, Cambridge, England, UK) was designed as a combination of two monoclonal antibodies that simultaneously bind to distinct non-overlapping epitopes of the viral spike protein and therefore exhibited broad coverage to variants (4–7). In addition, by reengineering Fc variant amino acids, the half-life of tixagevimab/cilgavimab was extended to approximately 90 days and the effectiveness in both pre-exposure prophylaxis and early outpatient treatment was demonstrated (5–7). Although the Omicron (B.1.1.529) variant and its subvariants, which contain numerous mutations at the spike protein regions, dominated the COVID-19 global outbreak in 2022, *in vitro* studies have demonstrated that tixagevimab/cilgavimab retains decreased, but still active neutralizing activity against the Omicron subvariants including BA.1, BA.2, BA.4, and BA.5 (4, 8–10). In South Korea, tixagevimab/cilgavimab has been prescribed for immunocompromised (IC) hosts who may not obtain sufficient vaccine-induced immunity from August 2022, when BA.5 became a dominant strain after a large outbreak wave of BA.1/BA.2. To estimate the clinical significance of tixagevimab/cilgavimab administration in the context of concurrent outbreak situations, we investigated serologic responses induced by tixagevimab/cilgavimab administration in IC hosts in comparison with those obtained from three-dose vaccinations, past infections from BA.1/BA.2, and recent infections from BA.5 among healthy adults.

## Methods

### Domestic outbreak situations and healthy adult sera for immunity level in population

**Figure 1:**
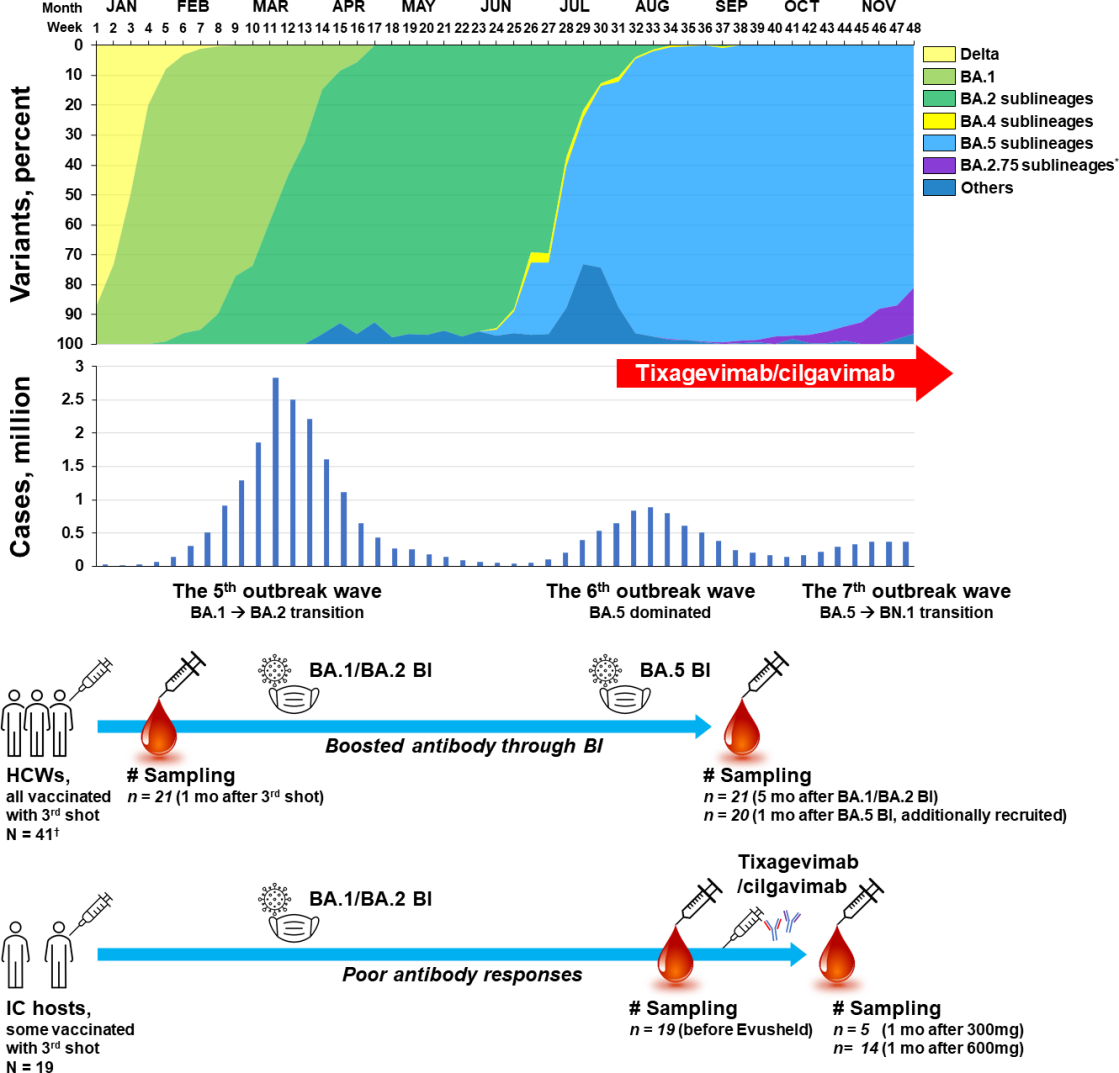
Schematic flow of sampling points presented with variant proportions and the number of domestic cases of COVID-19 per week in 2022, South Korea *Most of the BA.2.75 sublineages are reported to be BN.1. ^†^Twenty-one HCWs from a pre-existing booster shot cohort and 20 newly-recruited HCWs who experienced BA.5 BI. HCW, healthcare worker; IC, immunocompromised; BI, breakthrough infection; COVID-19, coronavirus disease 2019.

Information about domestic outbreak situation including the proportion of circulating SARS-CoV-2 variants and number of confirmed cases was collected from the weekly report of the Korea Disease Control and Prevention Agency (11). When a variant occupied more than half of the domestic strains, the variant was defined as a dominant strain. Accordingly, the BA.1/BA.2 outbreak period was corresponding to week 3 (January 2022) to week 29 (July 2022) (**Figure 1**). The transition from BA.1 to BA.2 occurred at week 12 (March 2022), but it was considered as a single BA.1/BA.2 outbreak period as the outbreak continued as one large wave (the 5^th^ domestic outbreak wave). BA.5 dominance was observed from week 30 (July 2022) and continued with two distinct outbreak waves (the 6^th^ and 7^th^ waves). During the 7^th^ wave, increase of the BA.2.75 sublineage (mainly BN.1) was observed from week 44 (November 2022). BA.5 sublineages such as BQ.1, BQ.1.1, and BF.7 were detected from week 41 (November 2022), but did not exceed 5% by November 2022.

For the evaluation of immunity level in healthy adult population when tixagevimab/cilgavimab was introduced in Korea, sera obtained from a heterologous booster shot cohort of healthcare workers (HCWs) were utilized (12). HCWs in this booster shot cohort received a 3^rd^ dose of mRNA COVID-19 vaccine (BNT162b2; Comirnaty^®^, Pfizer, NY, USA) after two standard doses of adenovirus vector (Adv) vaccines (ChAdOx1; Vaxzevria^®^, AstraZeneca, Oxford, UK). In the booster shot cohort, 21 HCWs who experienced breakthrough infections (BI) during the BA.1/BA.2 outbreak period were included in the present analysis. Sera sampled one month after the 3^rd^ shot (mid-December 2021) and five months after BA.1/BA.2 BI (late July 2022) were used. Follow-up sera from HCWs of the booster shot cohort who did not experience BI were not included in the present comparison, because the antibody titers and neutralizing activities would have waned and would be significantly lower than those of the sera collected one month after the 3^rd^ shot (12). Since a limited number of HCWs in the booster shot cohort experienced BI during the BA.5 outbreak period, 20 HCWs who received three doses of vaccines (adv/adv/mRNA) and experienced BA.5 BI (after week 32 when BA.5 accounted for more than 90% of domestic cases) were additionally recruited. Sampling was performed one month after BA.5 BI (early October 2022).

### IC cohort with poor antibody response

The indications for tixagevimab/cilgavimab administration in South Korea were those who have not been confirmed to be SARS-CoV-2 positive within seven days, who are ≥ 12 years of age, weigh ≥ 40 kg, and meet one of the following conditions: 1) those receiving immunosuppressive therapy or high-intensity chemotherapy, 2) those who have received hematopoietic stem cell transplant or chimeric antigen receptor (CAR)-T cell therapy within six months, 3) those who have received a solid organ transplant within one year, 4) those who have human immunodeficiency virus infection and are not expected to have CD^4^ T cell ≥ 200/mm^2^ with sufficient treatment, and 5) those who have primary immunodeficiency. IC hosts who met the above criteria for tixagevimab/cilgavimab administration and exhibited poor antibody response either to three doses of COVID-19 vaccinations or to SARS-CoV-2 infection were prospectively recruited. Poor antibody response was defined as anti-spike protein antibody (Sab) response < 3,719.0 BAU/mL, which is the lowest level of the booster shot HCW cohort measured one month after the 3^rd^ shot (n = 43, median 12,918.0 BAU/mL, IQR 8,989.0–17,304.0 BAU/mL, and range 3719.0–38,544.0 BAU/mL). The determination to receive 300 mg (one vial) or 600 mg (two vials) of tixagevimab/cilgavimab was solely the patient’s discretion. Blood sampling was conducted before and one month after tixagevimab/cilgavimab administration. Informed consent was obtained from all the participants and this study was approved by our local institutional review board (SMC 2020-03-113, 2021-01-165, and 2021-11-0506).

### Data collection, diagnosis of SARS-CoV-2 infection, and serologic tests

Data on the baseline characteristics of age, sex, height, weight, body mass index (BMI), body surface area (BSA), underlying disease, date and type of COVID-19 vaccination, and history of SARS-CoV-2 infection were collected. The diagnosis of SARS-CoV-2 infection was based on the positive result of either reverse transcription-polymerase chain reaction or antigen testing of respiratory specimens. For the semi-quantitative measurement of Sab, an electrochemiluminescence immunoassay kit (Elecsys Anti-SARS-CoV-2 S, Roche Diagnostics, Basel, Switzerland) was utilized. Ant-SARS-CoV-2 S antibody concentration ≥ 0.8 U/mL was considered positive, and the linear range was 0.4–250 U/mL (13). Automated dilution was performed for up to a 1:50 dilution in the cobas e analyzer, and an additional manual dilution of up to 1:200 was applied for the saturated specimens. A linear titer-correlation of the kit and plaque reduction neutralization test (PRNT) was presented in previous publications (14). To standardize binding assay results to the binding antibody units (BAU) recommended by the World Health Organization, a correction factor of 0.972 provided by the manufacturer was multiplied to the result (15, 16). To the evaluate neutralizing activity against BA.5, PRNT was conducted for the selected specimens (12, 17, 18). Considering the capacity of biosafety level-3 laboratory and time required for PRNT, 45 specimen were planned for PRNT. As binding assays were tested earlier, specimens with sufficient remaining volume were selected preferentially. SARS-CoV-2 dilutions to 40–50 PFU/well (BA.5 sublineage, Incheon, GRA clade, NCCP No. 43426) were prepared. Vero E6 cells were inoculated with serum and virus mixtures on a 12-well plate and incubated at 37°C, 5% CO_2_ for one hour. After the inoculums were removed, the cells were overlaid with 1 ml of modified Eagle’s medium (Gibco, Gaithersburg, MD, USA) containing 0.75% agarose and 2% fetal bovine serum (Gibco). The plates were incubated at 37°C with 5% CO_2_ for two or three days. Stain solution (0.07% crystal violet, 10% formaldehyde, and 5% ethanol) was then added to the cells, and the visualized plaques were counted. The 50% neutralizing dose (ND_50_) titer was calculated using the Karber formula (19).

### Statistical analysis

For comparative analysis of baseline characteristics, analysis of variance (ANOVA) and Tukey’s multiple comparisons test were used for continuous variables, and chi-square or Fisher’s exact tests were used for categorical variables. For the comparison of serologic tests, the Mann-Whitney U test was utilized. In the investigation of correlation between physical measurement values and antibody titers, a linear regression model was used. All *P* values were two-sided tests, and those < 0.050 were considered statistically significant. GraphPad Prism version 9.4.1 (GraphPad Software, San Diego, CA, USA) were used for all statistical analyses.

## Results

### Baseline characteristics of the study population

For the serologic investigation of tixagevimab/cilgavimab administration during the BA.5 outbreak period, a total of 19 IC hosts (five received tixagevimab/cilgavimab 300 mg and 14 received 600 mg) and 41 HCWs (21 who had experienced BA.1/BA.2 BI and 20 who had experienced BA.5 BI) were evaluated (**Figure 1**). **Table 1** shows the baseline characteristics of the patients. The average age of the IC hosts (53.9 **±** 13.8 years) was older than the HCWs with BA.1/BA.2 BI (43.3 **±** 9.1 years) or HCWs with BA.5 BI (43.2 ± 10.0 years). The sex distribution, BMI, and BSA were not significantly different between the groups. More patients in the IC hosts exhibited hypertension (36.8%) compared to the HCWs with BA.1/BA.2 BI (4.8%), while other underlying diseases except for the main causes of IC did not differ between the groups. All the HCWs received three doses of COVID-19 vaccine with Adv/Adv/mRNA vaccine schedules. Among the IC hosts, 13 patients (68.4%) received two or more doses of COVID-19 vaccines (two patients received four doses of vaccines, eight patients received three doses of vaccines, and three patients received two doses of vaccines), while six patients did not receive any vaccinations (31.6%). All non-vaccinated and two-dose-vaccinated IC hosts experienced SARS-CoV-2 infections. Poor antibody responses were determined by the samples taken either one month after three doses of vaccines or at the convalescent status of SARS-CoV-2 infection (< 3,719.0 BAU/mL).

**Table 1:** Baseline characteristics of the study population.

	IC hosts with poor antibody response (n = 19)	HCWs with BA.1/BA.2 BI (n = 21)	HCWs with BA.5 BI (n = 20)	*P* value
Demographics				
Age, year	53.9 **±** 13.8	43.3 **±** 9.1^*^	43.2 ± 10.0^*^	0.003
Male sex	13 (68.4)	9 (42.9)	8 (40.0)	0.149
BMI, kg/m^2^	23.2 ± 2.9	21.8 ± 2.4	23.6 ± 3.1	0.098
BSA^‡^, m^2^	1.5 ± 0.3	1.4 ± 0.3	1.6 ± 0.4	0.361
Underlying disease				
Hypertension	7 (36.8)	1 (4.8)^*^	3 (15.0)	0.029
Diabetes mellitus	1 (5.3)	0 (0.0)	0 (0.0)	0.334
Dyslipidemia	1 (5.3)	0 (0.0)	1 (5.0)	0.572
Chronic heart disease	3 (15.8)	0 (0.0)	1 (5.0)	0.127
Thyroid cancer	1 (5.3)	0 (0.0)	1 (5.0)	0.572
Diseases causing IC condition				
B-cell malignancy	9 (47.4)	none	none	NA
Lymphoma	8 (42.1)	none	none	NA
ALL	1 (5.3)	none	none	NA
Organ transplantation	9 (47.4)	none	none	NA
Kidney	8 (42.1)	none	none	NA
Heart	1 (5.3)	none	none	NA
Good’s syndrome	1 (5.3)	none	none	NA
Vaccination status				
Four doses				
Adv/Adv/mRNA/mRNA	2 (10.5)	0 (0.0)	0 (0.0)	0.107
Three doses	8 (42.1)	21 (100.0)^*^	20 (100.0)^*^	0.020
Adv/Adv/mRNA	1 (5.3)	21 (100.0)^*^	20 (100.0)^*^	<0.001
Adv/mRNA/mRNA	2 (10.5)	0 (0.0)	0 (0.0)	0.107
mRNA/mRNA/mRNA	5 (26.3)	0 (0.0)^*^	0 (0.0)^*^	0.003
Two doses				
mRNA/mRNA	3 (15.8)	0 (0.0)	0 (0.0)	0.033
None**^†^**	6 (31.6)	0 (0.0)^*^	0 (0.0)^*^	0.001
**SARS-CoV-2 infection**	10 (52.6)	21 (100.0)^*^	20 (100.0)^*^	<0.001
BA.1/BA.2 period	10 (52.6)	21 (100.0)^*^	0 (0.0)^*^	<0.001
BA.5 period	0 (0.0)	0 (0.0)	20 (100.0)^*^	<0.001

Data are expressed as the number (%) of patients or mean ± standard deviation. ^*^Significantly different in a two-group comparison with IC hosts. ^†^All non-vaccinated hosts experienced SARS-CoV-2 infection and exhibited poor antibody responses after convalescence. ^‡^BSA was calculated based on the Mosteller method.

IC, immunocompromised; HCW, healthcare worker; BI, breakthrough infection; BMI, body mass index; ALL, acute lymphoblastic leukemia; NA, not applicable; Adv, adenovirus vector vaccines; mRNA, messenger ribonucleic acid vaccines; SARS-CoV-2, severe acute respiratory syndrome coronavirus 2.

### Serologic response after tixagevimab/cilgavimab, three-dose vaccination, and BI

**Figure 2:**
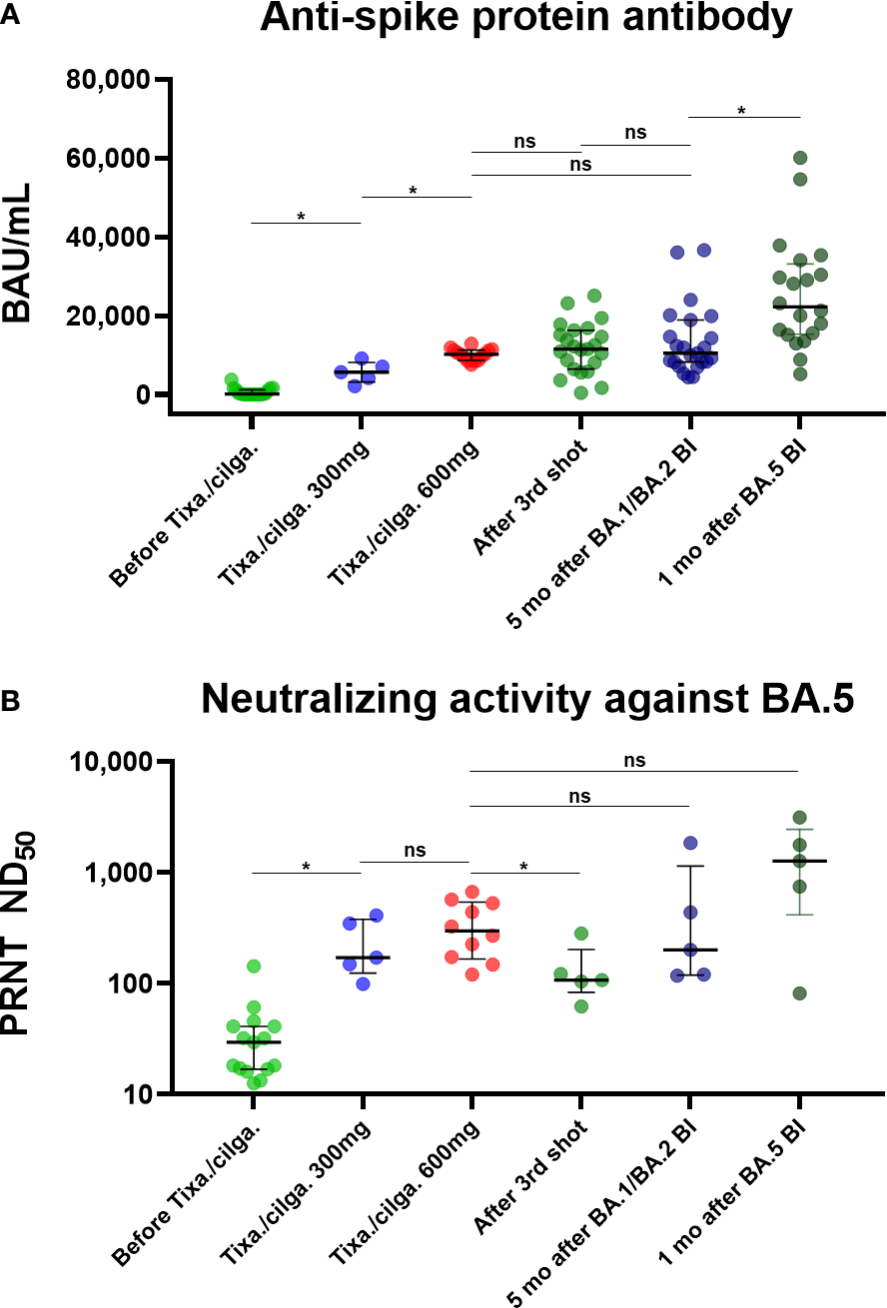
**(A)** Anti-spike protein antibody, presented as BAU/mL. **(B)** Neutralizing activity against BA.5, presented as PRNT ND_50_. Binding and neutralizing antibody responses after tixagevimab/cilgavimab in comparison with those to vaccination and BI ^*^Statistically significant difference was noticed. BI, breakthrough infection; BAU, binding antibody unit; tixa./cilga., tixagevimab/cilgavimab; PRNT, plaque reduction neutralization test; ND_50_, 50% neutralization dose; ns, not significant.

**Table 2** and **Figure 2** present the serologic response after tixagevimab/cilgavimab, in comparison with three-dose vaccination and BI. Sab was measured in all the collected specimens. All the tixagevimab/cilgavimab groups exhibited a statistically significant increase in Sab titers compared to before administration (median 130.2 BAU/mL before tixagevimab/cilgavimab, 5,665.8 BAU/mL after tixagevimab/cilgavimab 300 mg, 10,217 BAU/mL after tixagevimab/cilgavimab 600 mg, both *P* < 0.001; **Figure 2A**), and the titers significantly increased more when 600 mg was administered versus 300 mg (*P* = 0.002). Compared with tixagevimab/cilgavimab 600 mg administration, there was no statistically significant difference in the Sab titers one month after the 3^rd^ shot (12,144.2 BAU/mL, *P* = 0.175) or five months after BA.1 BI (10,455.8 BAU/mL, *P* = 0.778). In contrast, the titers measured one month after BA.5 BI (22,216.0 BAU/mL) were significantly higher than those after tixagevimab/cilgavimab 600 mg administration (*P* < 0.001). When compared with tixagevimab/cilgavimab 300 mg administration, Sab titers were significantly higher one month after the 3^rd^ shot, five months after BA.1 BI, and one month after BA.5 BI (*P* = 0.008, *P* = 0.019, and *P* < 0.001, respectively).

**Table 2:** Serologic responses to tixagevimab/cilgavimab in comparison with those to vaccination and BI.

Groups	Sampling point	Sab, BAU/mL	BA.5 PRNT, ND_50_
**IC hosts with poor antibody response** (n = 19)	**Before tixa./cilga.** *Binding ab test, n = 19* *PRNT, n = 15*	130.2 (0.4–1,165.4)	29.6 (17.0–41.2)
	**After tixa./cilga. 300 mg** *Binding ab test, n = 5* *PRNT, n = 5*	5,665.8 (3,162.9–8,105.0)	170.8 (124.2–379.0)
	**After tixa./cilga. 600 mg** *Binding ab test, n = 14* *PRNT, n = 10*	10,216.7 (8,602.2–11,274.2)	298.5 (166.9–540.8)
**HCWs with BA.1/BA.2 BI** (n = 21)	**1 mo after 3^rd^ shot** *Binding ab test, n = 21* *PRNT, n = 5*	12,144.2 (8,386.9–16,562.9)	107.6 (83.3–202.6)
	**5 mo after BA.1/BA.2 BI** *Binding ab test, n = 21* *PRNT, n = 5*	10,455.8 (7,679.8–19,418.6)	200.9 (118.9–1,144.4)
**HCWs with BA.5 BI** (n = 20)	**1 mo after BA.5 BI** *Binding ab test, n = 20* *PRNT, n = 5*	22,216.0 (15,272.1–33,148.1)	1,272.5 (415.3–2,448.7)

Data are expressed as median (IQR).

BI, breakthrough infection; Sab, anti-spike protein antibody; BAU, binding antibody unit; PRNT, plaque reduction neutralization test; ND_50_, 50% neutralization dose; IC, immunocompromised; tixa./cilga., tixagevimab/cilgavimab; HCW, healthcare worker; ab, antibody; mo, month; IQR, interquartile range.

A total of 45 specimens underwent PRNT against BA.5. Both tixagevimab/cilgavimab 300 mg and 600 mg induced a statistically significant increase in BA.5 PRNT ND_50_ than before administration (median ND_50_ 29.6 before tixagevimab/cilgavimab, 170.8 after tixagevimab/cilgavimab 300 mg, and 298.5 after tixagevimab/cilgavimab 600 mg, both *P* < 0.001; **Figure 2B**). A statistically significant difference was not noticed between tixagevimab/cilgavimab 300 mg and 600 mg (*P* = 0.310). Compared with tixagevimab/cilgavimab 600 mg administration, there was no statistically significant difference in the values five months after BA.1 BI (ND_50_ 200.9, *P* = 0.514), while ND_50_ of one month after the 3^rd^ shot was significantly lower (ND_50_ 107.6, *P* = 0.019). The ND_50_ of one month after BA.5 BI (ND_50_ 1,272.5) was highest among tested groups, but statistical difference was not noticed with tixagevimab/cilgavimab 600 mg (*P* = 0.151). When compared with tixagevimab/cilgavimab 300 mg administration, BA.5 PRNT ND_50_ titers were not significantly different with those measured one month after the 3^rd^ shot, five months after BA.1 BI, and one month after BA.5 BI (*P* = 0.222, *P* = 0.691, *P =* 0.151, respectively).

**Figure 3:**
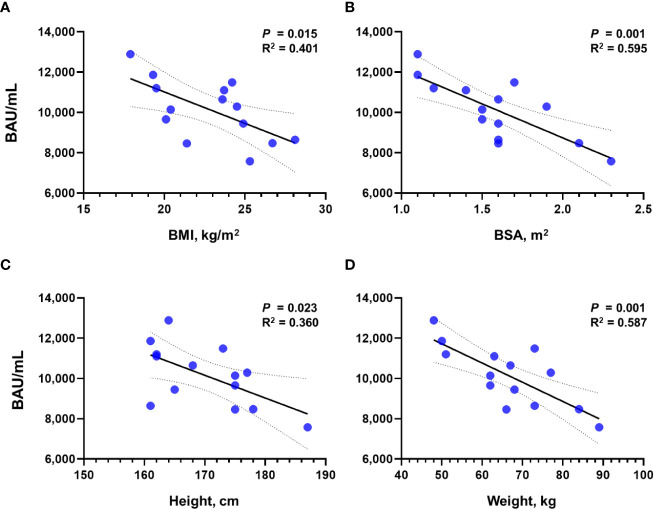
Correlation of Sab titers with physical measurement values after the administration of tixagevimab/cilgavimab 600 mg. Correlation with **(A)** BMI, **(B)** BSA, **(C)** height, and **(D)** weight is presented, respectively. Sab, anti-spike protein antibody; BMI, body mass index; BSA, body surface area; BAU, binding antibody unit.

To investigate the effect of physical characteristics on the effect of tixagevimab/cilgavimab, we calculated the correlation of Sab titers measured after the administration of tixagevimab/cilgavimab 600 mg with BMI, BSA, height, and weight (**Figure 3**). All physical characteristics demonstrated statistically significant reverse correlations with Sab (all *P* < 0.050), and the highest R^2^ value was noticed with BSA (R^2^ = 0.595). The BA.5 PRNT ND_50_ values also exhibited a reverse trend with physical measurement values, but statistical significances were not observed.

## Discussion

After the emergence of the Omicron variant with high transmissibility, approximately a half of the South Korean population experienced SARS-CoV-2 infection and IC hosts continue to be at risk of exposure to the virus (11, 20). As subvariants of Omicron succeeded the following outbreak waves, it became necessary to investigate the serologic status of the community together when estimating the serologic impact of monoclonal antibody agents to a circulating variant.

Of note, the administration of tixagevimab/cilgavimab 600 mg exhibited neutralizing activity against the BA.5 subvariant comparable with healthy adults who experienced BA.1/BA.2 BI, and higher than those vaccinated with the 3^rd^ dose. Those who received tixagevimab/cilgavimab 300 mg also exhibited comparable neutralizing activity with these comparators. PRNT ND_50_ of 118.25 has been suggested as the 50% protective neutralizing titer in our previous study (12). Since most patients exhibited BA.5 PRNT ND_50_ over 118.25 after tixagevimab/cilgavimab administration except for one (ND_50_ 99.4), the protective role of tixagevimab/cilgavimab is anticipated during the BA.5 dominant outbreak period. This result is in line with other study which suggested tixagevimab/cilgavimab maintained the neutralization activity against BA.5 in spite of a 30.7 fold reduction compared to against the ancestral strain (10). To date, several real-world studies suggested that pre-exposure tixagevimab/cilgavimab administration would be effective in IC hosts during the early Omicron period, but these studies did not reflect the emergence and outbreak of the BA.5 subvariant (21, 22). Our serologic investigation supports the continued use of tixagevimab/cilgavimab during BA.5 predominance, and additional cohort study would be required to evaluate the clinical effects at this period.

In another study by Benotmane et al., evaluating serologic effect of tixagevimab/cilgavimab administration among kidney transplant recipients, only 9.5% obtained neutralizing activity against the BA.1 subvariant after the administration of tixagevimab/cilgavimab 300 mg, and there was no neutralizing activity when Sab titers < 2,500 BAU/ml after tixagevimab/cilgavimab administration (23). In the present study, only one patient exhibited low Sab titer of 2145.2 BAU/mL and showed the lowest PRNT titer of ND_50_ 99.4. Benotmane et al. suggested that low tixagevimab/cilgavimab-induced antibody titers would be associated with high BMI. In the present study, we also investigated the correlation of tixagevimab/cilgavimab-induced antibody titers with physical characteristics and noticed that BSA exhibited the best reverse correlation with antibody titers. This finding may explain the finding of the present analysis that BA.5 PRNT ND_50_ titers obtained by tixagevimab/cilgavimab 300 mg administration were not significantly different from those after tixagevimab/cilgavimab 600 mg, as patients who received 300 mg had lower average BSA (1.4 **±** 0.2 m^2^) than those who received 600 mg (1.6 **±** 0.3 m^2^). Additional studies about the optimal dosage of monoclonal antibody agents according to body measurements should be conducted.

This study has several limitations. First, the present study was conducted with a relatively small cohort, which likely affected statistical significance. However, we tried to enroll a typical population that may represent the immune status of the community at the time period of tixagevimab/cilgavimab administration, and the present study may provide important insight into the serologic implications of tixagevimab/cilgavimab administration during the BA.5 outbreak period. Second, the study focused on serologic analysis and actual preventive effect of tixagevimab/cilgavimab was not evaluated. Since the dominant SARS-CoV-2 variant of each outbreak wave changes rapidly and the evaluation of preventive effect for the changing variant requires a considerable time, it would be important to investigate neutralizing activity of a monoclonal antibody agent against the dominant variant in comparison with the immune status of the community. In this context, our serologic study findings supported the administration of tixagevimab/cilgavimab during the BA.5 outbreak period in South Korea. Third, the present study does not provide information about how tixagevimab/cilgavimab would provide preventive effect for severe disease. Unlike active vaccination or natural infection that induce T cell immunity and B cell memory (24, 25), the role of passive immunization in preventing severe disease is still uncertain. To answer this question, clinical cohort study need to be conducted for IC hosts who were infected after tixagevimab/cilgavimab administration in comparison with those infected without tixagevimab/cilgavimab. Finally, since the measurement of antibody titers was only performed one month after tixagevimab/cilgavimab administration and was limited to neutralization of the BA.5 variant, additional studies evaluating waning time points and newly emerging variants such as BA.5 subvariants (e.g. BQ.1, BQ.1.1, or BF.7) and BA.2.75 subvariants (e.g. BN.1) or recombinants (e.g. XBB) are necessary.

In conclusion, tixagevimab/cilgavimab provided a comparable neutralizing activity against the BA.5 subvariant with those in a healthy adult population who had previous experiences with BA.1/BA.2 BI after three-dose vaccinations.

## Data availability statement

The original contributions presented in the study are included in the article/supplementary material. Further inquiries can be directed to the corresponding authors.

## Ethics statement

The studies involving human participants were reviewed and approved by the Institutional Review Board of Samsung Medical Center. The patients/participants provided their written informed consent to participate in this study.

## Author contributions

JY, BK, Y-kL, and J-HK were involved in the design of this study. JY, YL, HK, KH, SC, C-IK, DC, KP, KL, JP, SY, SK, WK, and J-HK enrolled the participants and collected specimens. GW, JB, MY, KK, and SH performed the experiments. JY, GW, JB, and J-HK assembled the data. JY, GW, KP, Y-kL, and J-HK were involved in the writing. All authors contributed to the article and approved the submitted version.
